# Bis(1,10-phenanthroline-κ^2^ *N*,*N*′)(sulfato-*O*)copper(II) butane-2,3-diol monosolvate

**DOI:** 10.1107/S1600536812049951

**Published:** 2012-12-12

**Authors:** Kai-Long Zhong, Guo-Qing Cao

**Affiliations:** a Department of Applied Chemistry, Nanjing College of Chemical Technology, Nanjing 210048, People’s Republic of China

## Abstract

The title compound, [Cu(SO_4_)(C_12_H_8_N_2_)_2_]·C_4_H_10_O_2_, is comprised of neutral monomeric complex and butane-2,3-diol solvent mol­ecules. In the complex, the Cu^II^ ion is in a distorted square-pyramidal coordination environment defined by four N atoms from two chelating 1,10-phenanthroline ligands and one O atom from a monodentate sulfate anion; the O atom is at the apex. The two chelating N_2_C_2_ groups subtend a dihedral angle of 85.8 (4)°. In the crystal, the neutral monomeric complex and butane-2,3-diol solvent mol­ecules are held together by O—H⋯O hydrogen bonding, which leads to additional stabilization of the structure. The presence of pseudosymmetry in the structure suggests the higher symmetry space group *C*2/*c*, but attempts to refine the structure in this space group resulted in an unsatisfactory model and high *R* and *wR* values. The sulfate anion is disordered over two sets of sites with occupancies of 0.55 (1) and 0.45 (1).

## Related literature

For the ethane-1,2-diol solvate of the title complex, see: Zhong (2011*a* ▶), for the propane-1,2-diol solvate, see: Zhong (2011*b* ▶) and for the propane-1,3-diol solvate, see: Zhong (2012 ▶). For related structures of transition metal complexes with a sulfate anion, see: Wang & Zhong (2011 ▶); Zhong & Ni (2012 ▶); Cui *et al.* (2010 ▶); Lu *et al.* (2006 ▶). 
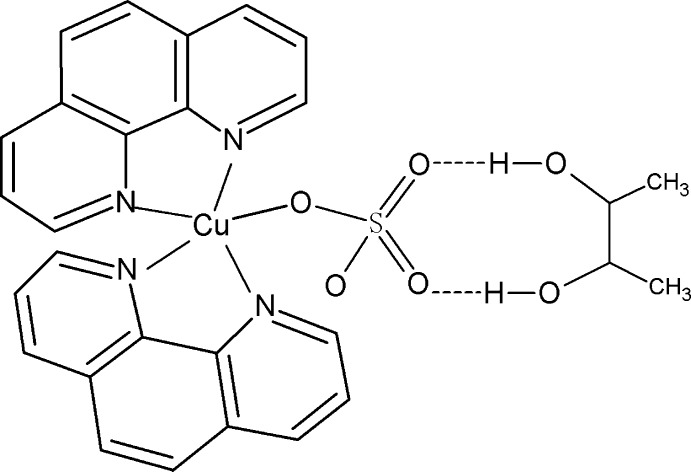


## Experimental

### 

#### Crystal data

[Cu(SO_4_)(C_12_H_8_N_2_)_2_]·C_4_H_10_O_2_
*M* *_r_* = 610.13
Monoclinic, 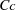

*a* = 17.352 (4) Å
*b* = 13.070 (3) Å
*c* = 13.444 (3) Å
β = 123.84 (3)°
*V* = 2532.4 (13) Å^3^
*Z* = 4
Mo *K*α radiation
μ = 1.00 mm^−1^
*T* = 223 K
0.32 × 0.27 × 0.21 mm

#### Data collection

Rigaku Mercury CCD diffractometer
Absorption correction: multi-scan (*REQAB*; Jacobson, 1998 ▶) *T*_min_ = 0.741, *T*_max_ = 0.818
7154 measured reflections
4178 independent reflections
3542 reflections with *I* > 2σ(*I*)
*R*_int_ = 0.026

#### Refinement

*R*[*F* ^2^ > 2σ(*F* ^2^)] = 0.046
*wR*(*F* ^2^) = 0.135
*S* = 0.99
4178 reflections
408 parameters
124 restraints
H-atom parameters constrained
Δρ_max_ = 0.60 e Å^−3^
Δρ_min_ = −0.53 e Å^−3^
Absolute structure: Flack (1983 ▶), 1317 Friedel pairs
Flack parameter: 0.55 (2)

### 

Data collection: *CrystalClear* (Rigaku, 2007 ▶); cell refinement: *CrystalClear* (Rigaku, 2007 ▶); data reduction: *CrystalClear* (Rigaku, 2007 ▶); program(s) used to solve structure: *SHELXS97* (Sheldrick, 2008 ▶); program(s) used to refine structure: *SHELXL97* (Sheldrick, 2008 ▶); molecular graphics: *XP* in *SHELXTL* (Sheldrick, 2008 ▶); software used to prepare material for publication: *SHELXTL* (Sheldrick, 2008 ▶).

## Supplementary Material

Crystal structure: contains datablock(s) global, I. DOI: 10.1107/S1600536812049951/bq2380sup1.cif

Structure factors: contains datablock(s) I. DOI: 10.1107/S1600536812049951/bq2380Isup2.hkl

Additional supplementary materials:  crystallographic information; 3D view; checkCIF report

## Figures and Tables

**Table 1:** Selected bond lengths (Å)

Cu1—O1	1.922 (11)
Cu1—O1′	1.944 (10)
Cu1—N1	2.000 (7)
Cu1—N4	2.014 (7)
Cu1—N3	2.091 (6)
Cu1—N2	2.186 (7)

**Table 2:** Hydrogen-bond geometry (Å, °)

*D*—H⋯*A*	*D*—H	H⋯*A*	*D*⋯*A*	*D*—H⋯*A*
O5—H5*B*⋯O3	0.82	1.92	2.73 (2)	172
O5—H5*B*⋯O4′	0.82	2.01	2.83 (2)	176
O6—H6⋯O3′	0.82	2.19	2.919 (16)	148
O6—H6⋯O4	0.82	1.95	2.720 (14)	156

**Fig. 1.**
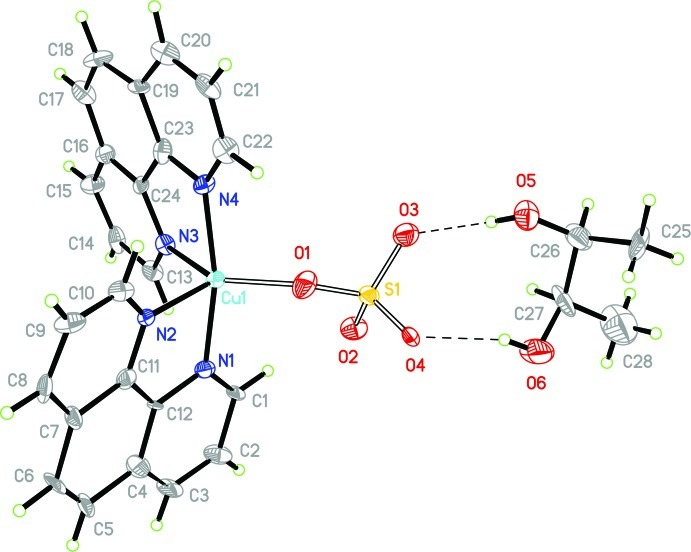
The molecular structure showing the atom-numbering scheme and with displacement ellipsoids drawn at the 35% probability level. Hydrogen bonds O—H···O are shown as dashed lines.

## supplementary crystallographic information

### Comment

In the past few years, we have unexpectedly obtained and characterized some transition metal complexes with bidentate-chelating sulfate auxiliary ligand *via* alcohol-solvothermal reaction during attempts to synthesize mixed-ligand coordination polymers, such as cobalt complex (Wang & Zhong, 2011), nickel complex (Zhong & Ni, 2012), zinc complex (Cui *et al.*, 2010), cadmium complex (Lu *et al.*, 2006). The title compound [CuSO_4_(C_12_H_8_N_2_)_2_].C_4_H_10_O_2_, (I), was obtained by the similar alcohol-solvothermal reaction and its crystal structure has not hitherto been reported.

The X-ray diffraction experiment found that the title complex is isotypical to the previously reported [CuSO_4_(C_12_H_8_N_2_)_2_].C_2_H_6_O_2_ (Zhong, 2011*a*), (II), [CuSO_4_(C_12_H_8_N_2_)_2_].CH_2_OHCHOHCH_3_ (Zhong, 2011*b*), (III), and [CuSO_4_(C_12_H_8_N_2_)_2_].CH_2_OHCH_2_CH_2_OH (Zhong, 2012), (IV). The geometry of the phenanthroline and sulfate ligands are in good agreement with those reported in the (II), (III) and (IV). The Cu^II^ metal ion is five-coordinated by four N atoms from two chelating phen ligands and an O atoms from a monodentate sulfate ligand, resulting in a distorted CuN_4_O square-pyramidal environment. The N1, N2, N3 and N4 atoms comprise a square, and the O1 atom site the apex of a square pyramid surrounding each metal atom (Fig 1). The dihedral angle of the two chelating N2C2 groups is 85.8 (4)°, which is larger than those reported in (II) [71.1 (2)°], (III) [84.9 (4)°] and (IV) [71.10 (15) Å], respectively. The Cu—O bond distance [1.922 (11) Å - 1.944 (10) Å], the Cu—N bond distance [2.000 (7) - 2.186 (7) Å], and the N—Cu—N bite angle [79.8 (3) - 81.6 (3)°] are comparable to those observed in (II), (III) and (IV) (Table 1).

In the crystal, the sulfate group is disordered over two positions with refined site occupancies of 0.55 (1) and 0.45 (1), and is hydrogen bonded to the solvent butane-2,3-diol molecule (Table 2 & Fig. 1).

### Experimental

The single crystals of (I) suitable to X-ray analysis were obtained by 0.2 mmol phen, 0.1 mmol CuSO_4_.5H_2_O, 2.0 ml propane-1,3-diol and 1.0 ml water mixed and placed in a thick Pyrex tube, which was sealed and heated to 453 K for 72 h.

### Refinement

The H atoms of phen were positioned geometrically and allowed to ride on their parent atoms, with C—H = 0.93 Å and *U*_iso_(H) = 1.2*U*_eq_(C). The H atoms of propane-1,3-diol were placed in geometrically idealized positions and refined as riding atoms, with C—H(CH_3_) = 0.96 Å, C—H(CH) = 0.98 Å and O—H = 0.82 Å; *U*_iso_(H) = 1.2*U*_eq_(C) and 1.5*U*_eq_(O). The presence of pseudo-symmetry in the structure suggests a higher symmetry space group *C*2/*c*. But attempts to refine the structure in the space group *C*2/*c* resulted in an unsatisfactory model and high *R* and *wR* values. Hence the requirement to solve in *Cc*. The reported Flack parameter was refined as s full least-squares and obtained by TWIN/BASF procedure in *SHELXL* (Sheldrick, 2008).

### Crystal data

**Table:**

[Cu(SO_4_)(C_12_H_8_N_2_)_2_]·C_4_H_10_O_2_	*F*(000) = 1260
*M**_r_* = 610.13	*D*_x_ = 1.600 Mg m^−^^3^
Monoclinic, *C**c*	Mo *K*α radiation, λ = 0.71073 Å
Hall symbol: C -2yc	Cell parameters from 5509 reflections
*a* = 17.352 (4) Å	θ = 3.2–27.5°
*b* = 13.070 (3) Å	µ = 1.00 mm^−^^1^
*c* = 13.444 (3) Å	*T* = 223 K
β = 123.84 (3)°	Block, green
*V* = 2532.4 (13) Å^3^	0.32 × 0.27 × 0.21 mm
*Z* = 4	

### Data collection

**Table:**

Rigaku Mercury CCD diffractometer	4178 independent reflections
Radiation source: fine-focus sealed tube	3542 reflections with *I* > 2σ(*I*)
Graphite Monochromator monochromator	*R*_int_ = 0.026
Detector resolution: 28.5714 pixels mm^-1^	θ_max_ = 27.5°, θ_min_ = 3.3°
ω scans	*h* = −15→22
Absorption correction: multi-scan (REQAB; Jacobson, 1998)	*k* = −16→15
*T*_min_ = 0.741, *T*_max_ = 0.818	*l* = −17→16
7154 measured reflections	

### Refinement

**Table:**

Refinement on *F*^2^	Secondary atom site location: difference Fourier map
Least-squares matrix: full	Hydrogen site location: inferred from neighbouring sites
*R*[*F*^2^ > 2σ(*F*^2^)] = 0.046	H-atom parameters constrained
*wR*(*F*^2^) = 0.135	*w* = 1/[σ^2^(*F*_o_^2^) + (0.1*P*)^2^] where *P* = (*F*_o_^2^ + 2*F*_c_^2^)/3
*S* = 0.99	(Δ/σ)_max_ = 0.002
4178 reflections	Δρ_max_ = 0.60 e Å^−^^3^
408 parameters	Δρ_min_ = −0.53 e Å^−^^3^
124 restraints	Absolute structure: Flack (1983), 1317 Friedel pairs
Primary atom site location: structure-invariant direct methods	Flack parameter: 0.55 (2)

### Special details

**Table:**

Geometry. All e.s.d.'s (except the e.s.d. in the dihedral angle between two l.s. planes) are estimated using the full covariance matrix. The cell e.s.d.'s are taken into account individually in the estimation of e.s.d.'s in distances, angles and torsion angles; correlations between e.s.d.'s in cell parameters are only used when they are defined by crystal symmetry. An approximate (isotropic) treatment of cell e.s.d.'s is used for estimating e.s.d.'s involving l.s. planes.
Refinement. Refinement of *F*^2^ against ALL reflections. The weighted *R*-factor *wR* and goodness of fit *S* are based on *F*^2^, conventional *R*-factors *R* are based on *F*, with *F* set to zero for negative *F*^2^. The threshold expression of *F*^2^ > σ(*F*^2^) is used only for calculating *R*-factors(gt) *etc*. and is not relevant to the choice of reflections for refinement. *R*-factors based on *F*^2^ are statistically about twice as large as those based on *F*, and *R*- factors based on ALL data will be even larger.

### Fractional atomic coordinates and isotropic or equivalent isotropic displacement parameters (Å^2^)

**Table:**

	*x*	*y*	*z*	*U*_iso_*/*U*_eq_	Occ. (<1)
Cu1	0.26519 (10)	0.30074 (3)	0.29387 (13)	0.02436 (16)	
S1	0.2748 (3)	0.5417 (4)	0.2771 (4)	0.0265 (13)	0.547 (12)
S1'	0.2569 (3)	0.5422 (4)	0.3106 (4)	0.0170 (14)	0.453 (12)
O1	0.2897 (10)	0.4419 (8)	0.3416 (12)	0.043 (3)	0.547 (12)
O1'	0.2430 (10)	0.4435 (7)	0.2448 (11)	0.027 (3)	0.453 (12)
O2	0.2706 (8)	0.5188 (9)	0.1677 (9)	0.037 (3)	0.547 (12)
O2'	0.2653 (8)	0.5225 (8)	0.4213 (8)	0.023 (3)	0.453 (12)
O3	0.1922 (11)	0.5995 (16)	0.2536 (19)	0.041 (5)	0.547 (12)
O3'	0.3442 (10)	0.5860 (12)	0.3305 (17)	0.021 (3)	0.453 (12)
O4	0.3552 (9)	0.6066 (10)	0.3586 (11)	0.021 (3)	0.547 (12)
O4'	0.1754 (13)	0.6044 (16)	0.2259 (16)	0.027 (5)	0.453 (12)
O5	0.2174 (4)	0.7813 (3)	0.3686 (4)	0.0442 (11)	
H5B	0.2079	0.7299	0.3282	0.066*	
O6	0.3460 (5)	0.8062 (5)	0.2956 (8)	0.096 (3)	
H6	0.3455	0.7532	0.3279	0.144*	
N1	0.3506 (5)	0.2822 (5)	0.2394 (6)	0.0244 (14)	
N2	0.3706 (5)	0.2068 (4)	0.4426 (6)	0.0236 (15)	
N3	0.1647 (5)	0.2096 (5)	0.1528 (5)	0.0220 (14)	
N4	0.1742 (5)	0.2848 (5)	0.3410 (6)	0.0232 (14)	
C1	0.3444 (6)	0.3269 (7)	0.1484 (8)	0.0282 (17)	
H1A	0.3000	0.3772	0.1053	0.034*	
C2	0.4087 (7)	0.2967 (7)	0.1148 (9)	0.038 (2)	
H2A	0.4052	0.3285	0.0505	0.046*	
C3	0.4728 (6)	0.2229 (7)	0.1768 (8)	0.0375 (18)	
H3A	0.5100	0.2000	0.1517	0.045*	
C4	0.4826 (7)	0.1813 (8)	0.2797 (9)	0.038 (2)	
C5	0.5554 (7)	0.1082 (5)	0.3559 (8)	0.032 (2)	
H5A	0.5958	0.0858	0.3355	0.039*	
C6	0.5640 (6)	0.0738 (6)	0.4535 (8)	0.036 (2)	
H6A	0.6112	0.0273	0.5009	0.043*	
C7	0.5031 (6)	0.1051 (6)	0.4909 (8)	0.0281 (18)	
C8	0.5105 (7)	0.0762 (5)	0.5960 (9)	0.032 (2)	
H8A	0.5567	0.0309	0.6490	0.038*	
C9	0.4489 (6)	0.1151 (7)	0.6208 (8)	0.038 (2)	
H9A	0.4540	0.0982	0.6915	0.046*	
C10	0.3805 (6)	0.1789 (7)	0.5391 (7)	0.0277 (18)	
H10A	0.3384	0.2035	0.5554	0.033*	
C11	0.4289 (5)	0.1747 (6)	0.4123 (7)	0.0270 (18)	
C12	0.4198 (5)	0.2096 (5)	0.3075 (7)	0.0183 (15)	
C13	0.1532 (6)	0.1797 (6)	0.0470 (7)	0.0293 (19)	
H13A	0.1933	0.2046	0.0273	0.035*	
C14	0.0804 (6)	0.1109 (6)	−0.0329 (7)	0.032 (2)	
H14A	0.0736	0.0927	−0.1043	0.039*	
C15	0.0208 (6)	0.0714 (7)	−0.0069 (8)	0.033 (2)	
H15A	−0.0248	0.0245	−0.0575	0.040*	
C16	0.0311 (6)	0.1046 (6)	0.1007 (8)	0.0284 (18)	
C17	−0.0306 (6)	0.0719 (6)	0.1307 (7)	0.035 (2)	
H17A	−0.0777	0.0261	0.0808	0.042*	
C18	−0.0226 (6)	0.1067 (7)	0.2327 (8)	0.037 (2)	
H18A	−0.0630	0.0823	0.2521	0.045*	
C19	0.0465 (5)	0.1794 (6)	0.3089 (6)	0.0215 (15)	
C20	0.0547 (6)	0.2284 (8)	0.4077 (7)	0.0346 (18)	
H20A	0.0129	0.2132	0.4283	0.042*	
C21	0.1237 (7)	0.2981 (6)	0.4733 (9)	0.036 (2)	
H21A	0.1324	0.3270	0.5420	0.043*	
C22	0.1777 (6)	0.3233 (7)	0.4369 (6)	0.0324 (19)	
H22A	0.2228	0.3726	0.4816	0.039*	
C23	0.1077 (6)	0.2146 (6)	0.2782 (7)	0.0281 (18)	
C24	0.1010 (5)	0.1709 (6)	0.1728 (6)	0.0194 (15)	
C25	0.2642 (9)	0.9498 (6)	0.4054 (10)	0.059 (3)	
H25A	0.2309	0.9594	0.4425	0.089*	
H25B	0.2663	1.0133	0.3711	0.089*	
H25C	0.3262	0.9275	0.4643	0.089*	
C26	0.2142 (7)	0.8676 (6)	0.3052 (8)	0.053 (2)	
H26A	0.1498	0.8877	0.2462	0.063*	
C27	0.2654 (7)	0.8516 (5)	0.2453 (8)	0.058 (2)	
H27A	0.2238	0.8072	0.1771	0.069*	
C28	0.2703 (13)	0.9468 (10)	0.1881 (16)	0.101 (5)	
H28A	0.3013	0.9326	0.1487	0.151*	
H28B	0.3040	0.9982	0.2482	0.151*	
H28C	0.2086	0.9709	0.1304	0.151*	

### Atomic displacement parameters (Å^2^)

**Table:**

	*U*^11^	*U*^22^	*U*^33^	*U*^12^	*U*^13^	*U*^23^
Cu1	0.0249 (3)	0.0219 (2)	0.0321 (3)	−0.0008 (5)	0.0194 (2)	−0.0011 (5)
S1	0.026 (3)	0.027 (2)	0.029 (2)	−0.0005 (17)	0.017 (2)	−0.0049 (19)
S1'	0.017 (3)	0.014 (2)	0.017 (2)	−0.0005 (17)	0.008 (2)	−0.0029 (18)
O1	0.054 (5)	0.037 (5)	0.044 (5)	−0.006 (3)	0.031 (4)	0.005 (3)
O1'	0.045 (5)	0.009 (4)	0.029 (5)	0.001 (3)	0.023 (4)	0.004 (3)
O2	0.043 (5)	0.049 (5)	0.029 (4)	−0.002 (3)	0.025 (3)	−0.002 (3)
O2'	0.022 (4)	0.024 (4)	0.015 (4)	−0.009 (3)	0.006 (3)	0.001 (3)
O3	0.034 (6)	0.042 (6)	0.047 (7)	0.000 (4)	0.022 (4)	0.005 (4)
O3'	0.017 (5)	0.016 (5)	0.028 (5)	−0.001 (3)	0.011 (4)	−0.003 (4)
O4	0.017 (4)	0.019 (5)	0.021 (5)	0.004 (3)	0.007 (3)	−0.004 (3)
O4'	0.029 (6)	0.028 (6)	0.024 (6)	0.002 (4)	0.016 (4)	0.003 (4)
O5	0.066 (3)	0.038 (2)	0.041 (2)	0.001 (2)	0.037 (2)	−0.0007 (19)
O6	0.076 (4)	0.060 (4)	0.188 (7)	0.039 (3)	0.096 (5)	0.071 (4)
N1	0.023 (3)	0.026 (3)	0.027 (3)	−0.003 (3)	0.016 (3)	−0.002 (3)
N2	0.027 (3)	0.017 (3)	0.038 (4)	−0.001 (2)	0.025 (3)	0.000 (2)
N3	0.019 (3)	0.026 (3)	0.011 (3)	0.002 (2)	0.002 (2)	−0.001 (2)
N4	0.022 (3)	0.027 (3)	0.019 (3)	−0.008 (3)	0.010 (3)	−0.007 (3)
C1	0.033 (4)	0.026 (3)	0.047 (5)	0.009 (3)	0.036 (4)	0.008 (3)
C2	0.034 (4)	0.055 (5)	0.035 (4)	0.002 (3)	0.025 (4)	−0.009 (3)
C3	0.038 (4)	0.046 (4)	0.048 (4)	0.005 (3)	0.035 (4)	−0.011 (4)
C4	0.043 (5)	0.035 (4)	0.048 (5)	0.003 (4)	0.033 (4)	−0.007 (3)
C5	0.034 (5)	0.017 (3)	0.044 (5)	0.008 (3)	0.021 (4)	−0.004 (3)
C6	0.023 (4)	0.025 (4)	0.054 (5)	0.009 (3)	0.018 (4)	−0.016 (3)
C7	0.023 (4)	0.019 (3)	0.031 (4)	0.007 (3)	0.009 (3)	0.007 (3)
C8	0.029 (5)	0.015 (3)	0.033 (5)	−0.002 (3)	0.006 (4)	0.008 (3)
C9	0.034 (5)	0.053 (5)	0.039 (5)	−0.006 (4)	0.028 (4)	0.001 (4)
C10	0.026 (4)	0.034 (4)	0.026 (4)	−0.009 (4)	0.016 (4)	−0.014 (3)
C11	0.019 (4)	0.022 (3)	0.031 (4)	−0.006 (3)	0.008 (3)	−0.004 (3)
C12	0.013 (3)	0.016 (3)	0.034 (4)	0.001 (3)	0.018 (3)	−0.002 (3)
C13	0.031 (4)	0.030 (3)	0.026 (4)	−0.001 (4)	0.015 (4)	−0.013 (3)
C14	0.039 (5)	0.020 (3)	0.020 (4)	0.001 (3)	0.005 (4)	−0.009 (3)
C15	0.021 (5)	0.043 (4)	0.028 (4)	−0.005 (4)	0.009 (4)	−0.003 (4)
C16	0.022 (4)	0.030 (4)	0.029 (4)	0.007 (3)	0.012 (3)	0.015 (4)
C17	0.026 (4)	0.036 (4)	0.030 (4)	0.002 (3)	0.007 (4)	−0.013 (3)
C18	0.022 (4)	0.060 (5)	0.034 (5)	−0.005 (4)	0.018 (4)	0.009 (4)
C19	0.009 (3)	0.033 (3)	0.020 (3)	0.005 (3)	0.007 (3)	0.007 (3)
C20	0.029 (4)	0.051 (4)	0.019 (3)	0.012 (3)	0.010 (3)	0.005 (3)
C21	0.044 (5)	0.036 (4)	0.033 (4)	0.007 (3)	0.025 (4)	−0.013 (3)
C22	0.024 (4)	0.039 (4)	0.017 (4)	−0.001 (3)	0.001 (3)	−0.006 (3)
C23	0.033 (4)	0.023 (4)	0.019 (4)	0.001 (3)	0.008 (3)	0.003 (3)
C24	0.026 (4)	0.017 (3)	0.019 (3)	0.000 (3)	0.015 (3)	0.002 (3)
C25	0.104 (6)	0.036 (4)	0.074 (5)	−0.044 (4)	0.071 (5)	−0.042 (4)
C26	0.070 (5)	0.046 (4)	0.069 (5)	0.016 (4)	0.055 (5)	0.009 (4)
C27	0.104 (7)	0.031 (3)	0.082 (6)	0.038 (4)	0.079 (6)	0.028 (4)
C28	0.149 (10)	0.089 (8)	0.110 (8)	0.024 (7)	0.100 (7)	0.021 (6)

### Geometric parameters (Å, º)

**Table:**

Cu1—O1	1.922 (11)	C7—C11	1.445 (11)
Cu1—O1'	1.944 (10)	C8—C9	1.382 (14)
Cu1—N1	2.000 (7)	C8—H8A	0.9300
Cu1—N4	2.014 (7)	C9—C10	1.364 (12)
Cu1—N3	2.091 (6)	C9—H9A	0.9300
Cu1—N2	2.186 (7)	C10—H10A	0.9300
S1—O2	1.463 (10)	C11—C12	1.404 (12)
S1—O4	1.471 (12)	C13—C14	1.430 (11)
S1—O3	1.490 (15)	C13—H13A	0.9300
S1—O1	1.506 (11)	C14—C15	1.365 (14)
S1'—O2'	1.435 (10)	C14—H14A	0.9300
S1'—O4'	1.470 (15)	C15—C16	1.423 (14)
S1'—O3'	1.498 (14)	C15—H15A	0.9300
S1'—O1'	1.507 (10)	C16—C24	1.361 (12)
O5—C26	1.396 (9)	C16—C17	1.405 (12)
O5—H5B	0.8200	C17—C18	1.377 (12)
O6—C27	1.307 (9)	C17—H17A	0.9300
O6—H6	0.8200	C18—C19	1.423 (12)
N1—C1	1.306 (10)	C18—H18A	0.9300
N1—C12	1.399 (10)	C19—C20	1.408 (11)
N2—C10	1.265 (11)	C19—C23	1.412 (11)
N2—C11	1.350 (11)	C20—C21	1.366 (13)
N3—C24	1.372 (10)	C20—H20A	0.9300
N3—C13	1.379 (10)	C21—C22	1.318 (14)
N4—C23	1.341 (10)	C21—H21A	0.9300
N4—C22	1.352 (11)	C22—H22A	0.9300
C1—C2	1.472 (12)	C23—C24	1.472 (11)
C1—H1A	0.9300	C25—C26	1.555 (13)
C2—C3	1.352 (13)	C25—H25A	0.9600
C2—H2A	0.9300	C25—H25B	0.9600
C3—C4	1.406 (13)	C25—H25C	0.9600
C3—H3A	0.9300	C26—C27	1.510 (9)
C4—C12	1.384 (11)	C26—H26A	0.9800
C4—C5	1.455 (13)	C27—C28	1.490 (14)
C5—C6	1.314 (13)	C27—H27A	0.9800
C5—H5A	0.9300	C28—H28A	0.9600
C6—C7	1.460 (12)	C28—H28B	0.9600
C6—H6A	0.9300	C28—H28C	0.9600
C7—C8	1.398 (14)		
			
O1—Cu1—O1'	32.6 (2)	N2—C10—C9	124.3 (8)
O1—Cu1—N1	99.6 (4)	N2—C10—H10A	117.9
O1'—Cu1—N1	92.2 (4)	C9—C10—H10A	117.9
O1—Cu1—N4	93.5 (4)	N2—C11—C12	121.1 (7)
O1'—Cu1—N4	99.5 (4)	N2—C11—C7	119.1 (8)
N1—Cu1—N4	166.8 (3)	C12—C11—C7	119.7 (8)
O1—Cu1—N3	140.1 (4)	C4—C12—N1	121.5 (7)
O1'—Cu1—N3	109.3 (4)	C4—C12—C11	121.2 (7)
N1—Cu1—N3	90.7 (3)	N1—C12—C11	116.9 (7)
N4—Cu1—N3	79.8 (3)	N3—C13—C14	120.9 (8)
O1—Cu1—N2	108.5 (4)	N3—C13—H13A	119.6
O1'—Cu1—N2	139.1 (4)	C14—C13—H13A	119.6
N1—Cu1—N2	81.6 (3)	C15—C14—C13	121.6 (8)
N4—Cu1—N2	93.3 (3)	C15—C14—H14A	119.2
N3—Cu1—N2	111.08 (12)	C13—C14—H14A	119.2
O2—S1—O4	111.2 (9)	C14—C15—C16	117.5 (7)
O2—S1—O3	112.8 (11)	C14—C15—H15A	121.3
O4—S1—O3	105.5 (12)	C16—C15—H15A	121.3
O2—S1—O1	107.4 (7)	C24—C16—C17	120.5 (8)
O4—S1—O1	106.5 (8)	C24—C16—C15	118.5 (8)
O3—S1—O1	113.4 (12)	C17—C16—C15	121.0 (8)
O2'—S1'—O4'	113.7 (11)	C18—C17—C16	121.0 (8)
O2'—S1'—O3'	112.0 (9)	C18—C17—H17A	119.5
O4'—S1'—O3'	111.5 (12)	C16—C17—H17A	119.5
O2'—S1'—O1'	110.2 (7)	C17—C18—C19	121.0 (8)
O4'—S1'—O1'	104.4 (10)	C17—C18—H18A	119.5
O3'—S1'—O1'	104.3 (10)	C19—C18—H18A	119.5
S1—O1—Cu1	134.8 (9)	C20—C19—C23	115.4 (7)
S1'—O1'—Cu1	133.3 (8)	C20—C19—C18	125.8 (8)
C26—O5—H5B	109.5	C23—C19—C18	118.5 (7)
C27—O6—H6	109.5	C21—C20—C19	120.3 (8)
C1—N1—C12	120.6 (7)	C21—C20—H20A	119.9
C1—N1—Cu1	126.8 (6)	C19—C20—H20A	119.9
C12—N1—Cu1	112.6 (5)	C22—C21—C20	118.4 (8)
C10—N2—C11	121.6 (7)	C22—C21—H21A	120.8
C10—N2—Cu1	131.8 (6)	C20—C21—H21A	120.8
C11—N2—Cu1	106.6 (5)	C21—C22—N4	126.6 (8)
C24—N3—C13	115.4 (6)	C21—C22—H22A	116.7
C24—N3—Cu1	112.8 (5)	N4—C22—H22A	116.7
C13—N3—Cu1	131.8 (6)	N4—C23—C19	124.2 (8)
C23—N4—C22	114.9 (8)	N4—C23—C24	116.8 (8)
C23—N4—Cu1	115.1 (6)	C19—C23—C24	119.0 (7)
C22—N4—Cu1	129.3 (6)	C16—C24—N3	126.2 (7)
N1—C1—C2	119.2 (8)	C16—C24—C23	119.7 (7)
N1—C1—H1A	120.4	N3—C24—C23	113.9 (7)
C2—C1—H1A	120.4	C26—C25—H25A	109.5
C3—C2—C1	120.4 (9)	C26—C25—H25B	109.5
C3—C2—H2A	119.8	H25A—C25—H25B	109.5
C1—C2—H2A	119.8	C26—C25—H25C	109.5
C2—C3—C4	119.4 (8)	H25A—C25—H25C	109.5
C2—C3—H3A	120.3	H25B—C25—H25C	109.5
C4—C3—H3A	120.3	O5—C26—C27	112.2 (6)
C12—C4—C3	118.7 (8)	O5—C26—C25	102.5 (7)
C12—C4—C5	119.4 (9)	C27—C26—C25	110.4 (9)
C3—C4—C5	121.9 (9)	O5—C26—H26A	110.5
C6—C5—C4	119.9 (9)	C27—C26—H26A	110.5
C6—C5—H5A	120.1	C25—C26—H26A	110.5
C4—C5—H5A	120.1	O6—C27—C28	107.1 (9)
C5—C6—C7	123.2 (8)	O6—C27—C26	124.3 (7)
C5—C6—H6A	118.4	C28—C27—C26	112.2 (8)
C7—C6—H6A	118.4	O6—C27—H27A	103.6
C8—C7—C11	116.9 (8)	C28—C27—H27A	103.6
C8—C7—C6	126.6 (8)	C26—C27—H27A	103.6
C11—C7—C6	116.4 (8)	C27—C28—H28A	109.5
C9—C8—C7	119.8 (8)	C27—C28—H28B	109.5
C9—C8—H8A	120.1	H28A—C28—H28B	109.5
C7—C8—H8A	120.1	C27—C28—H28C	109.5
C10—C9—C8	118.3 (8)	H28A—C28—H28C	109.5
C10—C9—H9A	120.9	H28B—C28—H28C	109.5
C8—C9—H9A	120.9		
			
O2—S1—O1—Cu1	−23.9 (15)	C11—C7—C8—C9	−0.8 (12)
O4—S1—O1—Cu1	−143.0 (11)	C6—C7—C8—C9	177.9 (8)
O3—S1—O1—Cu1	101.4 (15)	C7—C8—C9—C10	1.9 (12)
O1'—Cu1—O1—S1	−14.2 (12)	C11—N2—C10—C9	−0.1 (13)
N1—Cu1—O1—S1	64.7 (12)	Cu1—N2—C10—C9	176.6 (6)
N4—Cu1—O1—S1	−116.4 (12)	C8—C9—C10—N2	−1.5 (13)
N3—Cu1—O1—S1	−38.2 (16)	C10—N2—C11—C12	−177.2 (7)
N2—Cu1—O1—S1	149.0 (11)	Cu1—N2—C11—C12	5.4 (8)
O2'—S1'—O1'—Cu1	−18.3 (14)	C10—N2—C11—C7	1.3 (11)
O4'—S1'—O1'—Cu1	−140.8 (13)	Cu1—N2—C11—C7	−176.1 (6)
O3'—S1'—O1'—Cu1	102.1 (13)	C8—C7—C11—N2	−0.8 (11)
O1—Cu1—O1'—S1'	−17.4 (12)	C6—C7—C11—N2	−179.6 (7)
N1—Cu1—O1'—S1'	−121.9 (12)	C8—C7—C11—C12	177.7 (7)
N4—Cu1—O1'—S1'	64.2 (12)	C6—C7—C11—C12	−1.1 (11)
N3—Cu1—O1'—S1'	146.6 (11)	C3—C4—C12—N1	−3.4 (13)
N2—Cu1—O1'—S1'	−42.2 (15)	C5—C4—C12—N1	177.7 (7)
O1—Cu1—N1—C1	−65.3 (8)	C3—C4—C12—C11	−176.6 (8)
O1'—Cu1—N1—C1	−33.4 (8)	C5—C4—C12—C11	4.5 (12)
N4—Cu1—N1—C1	119.4 (13)	C1—N1—C12—C4	−1.3 (12)
N3—Cu1—N1—C1	76.0 (7)	Cu1—N1—C12—C4	176.2 (6)
N2—Cu1—N1—C1	−172.8 (8)	C1—N1—C12—C11	172.3 (8)
O1—Cu1—N1—C12	117.4 (6)	Cu1—N1—C12—C11	−10.3 (8)
O1'—Cu1—N1—C12	149.3 (6)	N2—C11—C12—C4	176.3 (8)
N4—Cu1—N1—C12	−57.9 (17)	C7—C11—C12—C4	−2.2 (11)
N3—Cu1—N1—C12	−101.3 (5)	N2—C11—C12—N1	2.8 (10)
N2—Cu1—N1—C12	10.0 (5)	C7—C11—C12—N1	−175.7 (7)
O1—Cu1—N2—C10	77.2 (8)	C24—N3—C13—C14	−1.5 (10)
O1'—Cu1—N2—C10	91.0 (9)	Cu1—N3—C13—C14	176.2 (5)
N1—Cu1—N2—C10	174.6 (8)	N3—C13—C14—C15	−0.9 (12)
N4—Cu1—N2—C10	−17.6 (8)	C13—C14—C15—C16	2.7 (12)
N3—Cu1—N2—C10	−97.9 (7)	C14—C15—C16—C24	−2.0 (12)
O1—Cu1—N2—C11	−105.6 (6)	C14—C15—C16—C17	176.5 (8)
O1'—Cu1—N2—C11	−91.8 (7)	C24—C16—C17—C18	0.6 (13)
N1—Cu1—N2—C11	−8.2 (5)	C15—C16—C17—C18	−177.8 (8)
N4—Cu1—N2—C11	159.6 (5)	C16—C17—C18—C19	2.1 (13)
N3—Cu1—N2—C11	79.2 (6)	C17—C18—C19—C20	172.6 (9)
O1—Cu1—N3—C24	−94.2 (8)	C17—C18—C19—C23	−0.5 (12)
O1'—Cu1—N3—C24	−107.6 (6)	C23—C19—C20—C21	−5.8 (12)
N1—Cu1—N3—C24	159.8 (5)	C18—C19—C20—C21	−179.1 (8)
N4—Cu1—N3—C24	−11.0 (5)	C19—C20—C21—C22	4.8 (13)
N2—Cu1—N3—C24	78.6 (6)	C20—C21—C22—N4	−2.1 (14)
O1—Cu1—N3—C13	88.0 (9)	C23—N4—C22—C21	0.6 (13)
O1'—Cu1—N3—C13	74.6 (8)	Cu1—N4—C22—C21	−168.9 (7)
N1—Cu1—N3—C13	−18.0 (7)	C22—N4—C23—C19	−1.9 (12)
N4—Cu1—N3—C13	171.2 (7)	Cu1—N4—C23—C19	169.2 (6)
N2—Cu1—N3—C13	−99.2 (7)	C22—N4—C23—C24	179.7 (7)
O1—Cu1—N4—C23	151.3 (7)	Cu1—N4—C23—C24	−9.2 (9)
O1'—Cu1—N4—C23	119.0 (7)	C20—C19—C23—N4	4.5 (12)
N1—Cu1—N4—C23	−33.4 (18)	C18—C19—C23—N4	178.3 (8)
N3—Cu1—N4—C23	10.9 (5)	C20—C19—C23—C24	−177.2 (7)
N2—Cu1—N4—C23	−99.9 (6)	C18—C19—C23—C24	−3.4 (11)
O1—Cu1—N4—C22	−39.2 (8)	C17—C16—C24—N3	−179.0 (7)
O1'—Cu1—N4—C22	−71.4 (8)	C15—C16—C24—N3	−0.6 (12)
N1—Cu1—N4—C22	136.2 (13)	C17—C16—C24—C23	−4.6 (12)
N3—Cu1—N4—C22	−179.6 (8)	C15—C16—C24—C23	173.9 (8)
N2—Cu1—N4—C22	69.6 (8)	C13—N3—C24—C16	2.3 (11)
C12—N1—C1—C2	2.7 (12)	Cu1—N3—C24—C16	−175.9 (6)
Cu1—N1—C1—C2	−174.3 (6)	C13—N3—C24—C23	−172.4 (7)
N1—C1—C2—C3	0.6 (13)	Cu1—N3—C24—C23	9.4 (8)
C1—C2—C3—C4	−5.2 (13)	N4—C23—C24—C16	−175.6 (7)
C2—C3—C4—C12	6.5 (14)	C19—C23—C24—C16	6.0 (11)
C2—C3—C4—C5	−174.7 (8)	N4—C23—C24—N3	−0.5 (10)
C12—C4—C5—C6	−3.6 (13)	C19—C23—C24—N3	−178.9 (7)
C3—C4—C5—C6	177.6 (9)	O5—C26—C27—O6	−42.6 (15)
C4—C5—C6—C7	0.2 (13)	C25—C26—C27—O6	71.1 (12)
C5—C6—C7—C8	−176.6 (9)	O5—C26—C27—C28	−174.2 (10)
C5—C6—C7—C11	2.0 (12)	C25—C26—C27—C28	−60.6 (10)

### Hydrogen-bond geometry (Å, º)

**Table:**

*D*—H···*A*	*D*—H	H···*A*	*D*···*A*	*D*—H···*A*
O5—H5*B*···O3	0.82	1.92	2.73 (2)	172
O5—H5*B*···O4′	0.82	2.01	2.83 (2)	176
O6—H6···O3′	0.82	2.19	2.919 (16)	148
O6—H6···O4	0.82	1.95	2.720 (14)	156
